# An unusual cause of ascites: Castleman disease

**DOI:** 10.1186/s12876-021-02050-7

**Published:** 2021-12-16

**Authors:** XiuLi Zhu, Si Chen, Fang Fang, Yong Jia, KaiGuang Zhang

**Affiliations:** Department of Gastroenterology, The First Affiliated Hospital of USTC, Division of Life Sciences and Medicine, University of Science and Technology of China, Hefei, 230001 Anhui People’s Republic of China

**Keywords:** Castleman disease, Ascites, Lymph nodes, Polyserositis, Case report

## Abstract

**Background:**

Castleman disease (CD) is a group of rare lymphoproliferative diseases with common lymph node histological features that can easily be misdiagnosed as infections, multiple autoimmune diseases, and malignant tumors.

**Case presentation:**

Here we report a rare case of a Chinese male with refractory ascites for two years and was eventually diagnosed as CD.

**Conclusions:**

The challenges in diagnosis of CD arise from the large differential, clinical heterogeneity and our limited understanding of pathology. In case of rare ascites, CD needs to be considered.

## Background

Castleman disease (CD), also known as lymphoid hamartoma or angiofollicular lymph node hyperplasia, was first described by Benjamin Castleman in 1954 [1]. It is a group of rare lymphoproliferative disorders with common lymph node histological features. The clinical manifestations of CD include lymphadenopathy, splenomegaly, hepatomegaly, anemia, skin and lung lesions, fluid accumulation, bleeding, infection, etc., of which lymphadenopathy is the main clinical feature [2]. Most of the patients go to the department of hematology or general surgery, and the final diagnosis is mostly in the department of hematology. Here we introduce a case of a patient who was diagnosed as CD in the gastroenterology department of our hospital due to refractory ascites. This case report provides a valuable reference for the diagnosis and treatment of CD and rare ascites.

## Case presentation

A 57-year-old man presented to the gastroenterology department of our hospital with refractory ascites for two years. He had no history of metabolic syndrome or alcohol consumption. He had a history of hypertension, hypothyroidism, and chronic nephritis, who was treated with nifedipine tablets and thyroxine tablets. He denied any fever, chest pain, rashes, oral ulcers, arthralgias and visual changes, and had no recent travel and no sick contacts. In the past two years, he has been treated in the gastroenterology department of many hospitals for ascites, and has undergone blood tests, ascites test, gastroscopy, colonoscopy, abdominal enhanced CT, etc. However, there was no clear diagnosis. The patients received oral or intravenous furosemide, oral spironolactone, and abdominal puncture drainage to resolve ascites in many hospitals, but the results were not satisfactory.

The physical examination included a poor general condition, palpable lymph nodes in both sides of the neck and groin with a larger diameter of about 1 cm, abdominal distension, no tenderness and rebound pain, positive mobile dullness, mild edema of both lower limbs, enlarged spleen which lower edge is 3 fingers under the ribs. The blood routine showed that white blood cells were 4.44 × 10^9^/L, hemoglobin was 111.0 g/L, and platelets were 93.0 × 10^9^/L. Urine protein was weakly positive, urine pentaprotein test showed that microalbumin was 82.40 mg/L (reference value 0–30 mg/L), immunoglobulin IgG was 33.40 mg/L (reference value 0–8.5 mg/L), transferrin was 3.29 mg/L (reference value 0–2.2 mg/L), α1-microglobulin was 54.20 mg/L (reference value 0–12 mg/L), β2-microglobulin was 0.19 mg/L (reference value 0–0.22 mg/L). Other positive laboratory indicators included uric acid 520 μmol/L, albumin 36.6 g/L, and erythrocyte sedimentation rate (ESR) 26.0 mm/h. Serum thyroid stimulating hormone (TSH) was 5.5400 mIU/L, serum free thyroxine (FT4) was 14.81 pmol/L, serum free triiodothyronine (FT3) was 1.74pmol/L, which was a slight decrease. Stool routine, urea nitrogen, creatinine, C-reactive protein (CRP), liver function, serum vitamin B12, IgG4, folic acid, hepatitis virus (A, B, C, D, E), tumor markers (CA125, CA199, CEA, AFP, PSA), brain natriuretic peptide (BNP), and tuberculosis detection (PPD test, T-spot), as well as other autoimmunity makers containing antinuclear antibody (ANA), anti-neutrophil cytoplasmic antibodies (ANCA), and rheumatoid factors were all unremarkable. The patient’s HIV, EBV, CMV or Toxoplasma was negative. HHV8 and IL-6 were not detected. The gastroscope showed superficial gastritis, and the colonoscopy showed no obvious abnormalities. The echocardiogram showed a little pericardial effusion. The enhanced CT of the chest and abdomen depicted pneumonia, bilateral pleural effusion, and abdominal effusion. We performed abdominal paracentesis for this patient. The ascites was yellow and clear, the nucleated cell count was 40 × 10^6^/L, the mononuclear cells accounted for 80.6%, and the multinucleated cells accounted for 19.4%. The Rivalta test was negative. The content of adenosine deaminase (ADA) in ascites was 2.6 U/L (reference value 0–25U/L), lactate dehydrogenase (LDH) was 74 IU/L (reference value 120–250 IU/L), albumin was 15.7 g/L, CA125 in ascites was 542 ng/mL (reference value 0–7 ng/mL), CEA, APF, and CA199 were normal. No malignant cells and tubercle bacilli were found in multiple tests of ascites. Serum ascitic albumin gradient (SAAG) was 20.9 g/L.

The patient had ascites, which should be polyserositis to be precise, superficial lymphadenopathy, enlarged spleen, hypothyroidism. We made differential diagnosis based on available data. The causes of ascites may be the following: liver cirrhosis, tuberculosis, tumor, rheumatism, endocrine, cardiac insufficiency, and nephritis. SAAG remains the most sensitive and specific marker for the differentiation of ascites due to portal hypertension from ascites due to other causes. The SAAG of the patient was greater than 11 g/L, however, there were no history of hepatitis, no esophageal/gastric varices under gastroscope, and no typical CT images of liver cirrhosis. We did not perform HVPG measurement and liver stiffness measurement, nor did we perform liver biopsy to rule out other rare causes of portal hypertension. We comprehensively considered and ruled out liver cirrhosis, which should be reported to a certain extent as a limitation of case reporting. He had no history or exposure of tuberculosis infection, no fever, no night sweats, negative tuberculosis test (PPD, T-spot), normal ADA in ascites, and no tuberculosis bacilli have been detected in ascites. So tuberculosis infection was also ruled out. The patient had a small amount of urine protein, mild hypothyroidism, normal rheumatism indicators, and no manifestation of cardiac insufficiency, so it was necessary to focus on tumors or other rare causes. After communicating with the patient and obtaining his consent, we gave him an in-depth comprehensive examination including bone marrow testing, PET-CT, and lymph node biopsy.

**Fig. 1:**
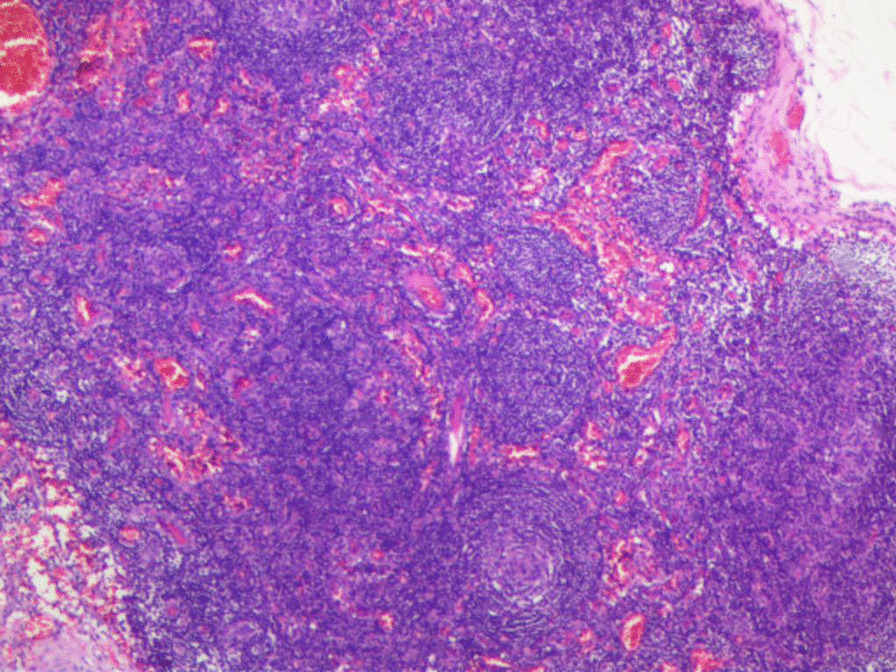
The lymph follicles increased, the germinal center was atrophied, the inter-follicular and paracortical areas showed vascular hyperplasia, and the mantle area was obviously hyperplasia with onion-skin-like change

PET-CT reported that his bilateral neck, axillary, retroperitoneum and groin had enlarged lymph nodes with a slight increase in FDG metabolism. Combined with the medical history, it was considered to be consistent with the metabolic changes of indolent lymphoma by the medical technicians. Bone marrow cytology indicated that bone marrow cells proliferated actively, granulocyte proliferation was obviously active with nucleus shifted to the right, erythroid proliferation was active, platelets were aggregated and distributed, and primitive cells accounted for about 1.0% of nuclear cells. The immunophenotyping of bone marrow lymphoma showed that the proportion of myeloid blasts was not high, with normal phenotype, the proportion of lymphocytes was not high, there were no abnormal monoclonal cells and no abnormal plasma cells. Was this patient with lymphoma? We were in confusion. Fortunately, the right neck lymph node biopsy pathology gave us the answer. Pathological examination of the lymph nodes showed that the lymph follicles increased, the germinal center was atrophied, the inter-follicular and paracortical areas showed vascular hyperplasia, and the mantle area was obviously hyperplasia with onion-skin-like change (Fig. 1). Onion-skin-like appearance was a typical pathological manifestation of CD. The immunohistochemical results were: CD3 (paracortical cells +), CD5 (paracortical cells +), CD20 (germinal center cells +), PAX5 (germinal center cells +), CD21 (follicular dendrites +), CD34 (Vascular +), Bcl-2 (mantle area +), SOX11 (−), Cyclin D1 (−), Ki-67 (+, about 10%). Finally, the patient was diagnosed with CD. We recommended him use CHOP chemotherapy, but he refused and chose oral thalidomide, the patient had poor compliance and refused to use steroid therapy. Three months later, his symptoms did not improve significantly. Due to economic reasons, he still refused chemotherapy and chose oral diuretics to relieve ascites.

## Discussion and conclusion

CD is clinically divided into unicentric CD (UCD), which is characterised by an asymptomatic mass in patients with one area of lymphadenopathy, and multicentric CD (MCD), which is characterised by constitutional symptoms, multicentric lymphadenopathy, hepatosplenomegaly and laboratory abnormalities such as anemia, hypergamma globulinemia, and bone marrow plasmacytosis [2]. The incidence of CD is very low. The estimated annual incidence of CD in the United States is 4300 to 5200 [3]. There are few epidemiological studies on CD. Male are slightly more often affected with MCD than female, but for UCD, there is no gender preference. The average age for diagnosis of UCD is usually younger (40 years) than MCD (60 years) [4, 5]. The diagnostic criteria of MCD include major criteria and minor criteria. Major criteria (both required): (1) histopathologic lymph node, (2) enlarged lymph nodes in ≥ 2 lymph node stations. Minor criteria (need ≥ 2 of 11 with ≥ 1 laboratory criterion): (1) laboratory: elevated ESR or CRP, anemia, thrombocytopenia/tosis, renal dysfunction or proteinuria, polyclonal hypergammaglobulinemia, hypoalbuminemia, (2) clinical: constitutional symptoms, large spleen and/or liver, fluid accumulation, eruptive cherry angiomata or violaceous papules, lymphocytic interstitial pneumonitis [2]. The typical pathological characteristics of CD are as follows: the mantle zone lymphocytes surrounding the follicles are arranged in concentric circles, showing a target-like shape. The broad, small and mature lymphocytes have concentrated chromatin with very little cytoplasm, presenting an onion-skin-like appearance. Usually, there may be radially penetrating sclerotic blood vessels that together with the amorphous follicles and concentric cloak areas impart a so-called “lollipop” appearance [6]. The cases described in our article meet the two main criteria of lymph node pathology and multiple lymph node enlargement, and meet the elevated ESR, anemia, proteinuria, hypoalbuminemia, constitutional symptoms, large spleen, and fluid accumulation in minor criteria. So the final diagnosis of the patient is MCD. There may be multiple mechanisms for the formation of ascites in this patient: (1) The compression of swollen lymph nodes in the abdominal cavity and retroperitoneum leads to blockage of the lymphatic vessels and fluid leakage. (2) Hypoalbuminemia and abnormal renal function cause the decrease of plasma colloidal osmotic pressure and water and sodium retention. There is no uniform conclusion about whether ascites is exudate or leaking fluid in this type of disease.

In MCD, the systemic manifestations and multiple areas of lymphadenopathy can look like multiple autoimmune diseases, acute infections, POEMS syndrome, and malignant tumors, especially lymphoma. The presence of positive diagnostic criteria, histopathologic lymph node, and enlarged lymph nodes assist with diagnosis. PET-CT was suspected to be lymphoma, but lymphoma was eventually ruled out through pathological examination of the lymph nodes. It is recommended to perform a biopsy at the site with the highest standardized uptake value (SUV) of the PET-CT scan, not only for obtaining a diagnostic sample but also for excluding lymphoma. The median maximum SUV for CD is usually 3 to 8, whereas higher values would suggest lymphoma [7]. We performed a biopsy at the right neck lymph node with SUV 8 which is a cut-off value, so we mistakenly believed that the diagnosis is lymphoma. The challenges in diagnosis of CD arise from thelarge differential, clinical heterogeneity and our limited understanding of pathology.

The first choice of treatment for UCD is surgical resection whenever possible. Complete surgical resection can cure almost all symptoms and return to normal laboratory abnormalities [8, 9]. The consensus guidance of van Rhee et al. outlines the recommendations for the treatment of MCD, including Siltuximab, Tocilizumab, Rituximab, corticosteroids, immunosuppressive agents, etc., but the efficacy is worse than UCD [4]. Unfortunately, the patient in this case could not receive first-line treatment due to economic reasons.

Although hematology specialists have a certain understanding of CD, there remains a lack of information among the non-specialists. Many patients go to gastroenterology or general internal medicine for ascites or polyserositis, and the knowledge of rare diseases among non-specialist seems to be insufficient. In case of rare ascites, CD needs to be considered.

## Data Availability

The datasets used and/or analysed during the current case reports are available from the corresponding author on reasonable request.

## Abbreviations

CD: Castleman disease
UCD: Unicentric CD
MCD: Multicentric CD
ESR: Erythrocyte sedimentation rate
TSH: Thyroid stimulating hormone
FT4: Free thyroxine
FT3: Free triiodothyronine
CRP: C-reactive protein
BNP: Brain natriuretic peptide
ANA: Antinuclear antibody
ANCA: Anti-neutrophil cytoplasmic antibodies
ADA: Adenosine deaminase
LDH: Lactate dehydrogenase
SAAG: Serum ascitic albumin gradient
SUV: Standardized uptake value
